# Integrating D–S evidence theory and multiple deep learning frameworks for time series prediction of air quality

**DOI:** 10.1038/s41598-025-87935-3

**Published:** 2025-02-18

**Authors:** Siling Feng, Le Tang, Mengxing Huang, Yuanyuan Wu

**Affiliations:** School of Information and Communication Engineering, Hainan University, No. 58 Renmin Avenue, Haikou, 570028 Hainan China

**Keywords:** Environmental sciences, Computer science

## Abstract

Accurate prediction of air quality time series data is helpful to identify and warn air pollution events in advance. Although the current air quality prediction models have made some progress in improving the accuracy of prediction, due to the impact of specific pollutants or complex meteorological conditions, these models still have the problems of low prediction accuracy, robustness and generalization ability in univariate prediction. In order to solve these problems, this study proposes a framework that integrates D–S evidence theory and a variety of deep learning models. The air quality data of three representative cities with climate characteristics in China are obtained and five indicators on air pollutants are collected. The preprocessed data are divided by time length to form short-term, medium-term and long-term input data, and MLP, RNN, CNN, LSTM, BI-LSTM and GRU models are established respectively. By comparing the performance indicators of the six models, three most suitable models are selected to predict the short, medium and long-term data respectively. Taking the prediction results and reliability as the three evidence bodies of the theory, a fusion model based on D–S evidence theory is established. For the three performance indicators MAE, RMSE and MAPE of the model, the best result of the fusion model increases the performance by 7.42%, 4.25% and 12.82% compared with the sub optimal architecture. This shows that integrating D–S evidence theory and a variety of deep learning algorithms provides an effective method to accurately predict the long-term air quality level in most urban areas.

## Introduction

In recent years, with the continuous deepening of urbanization and industrialization, the problem of air pollution in the urban environment has become increasingly serious, causing widespread concern in the community. The rapid growth of urban population and the improvement of the level of motorization have led to a sharp increase in traffic volume, which has led to a surge in exhaust emissions^1^. As an important indicator to measure the overall environmental quality of cities, air quality problems not only endanger the ecosystem, but also have a negative impact on human health, which has become a part of people’s livelihood^2^. Accurate prediction can help citizens better consider their own health and safety, so as to formulate reasonable travel planning. At present, although more efforts are being made to monitor and control air pollution, there are still many problems. In order to improve air quality, it is necessary to take systematic measures to establish a prediction mechanism for air pollutant concentration. However, there are many factors affecting air pollution, and the reasons are complex^3^. The existing research methods lack effective prediction of air pollution^4^.

Predicting the concentration of particulate matter in the atmosphere in advance is very important for taking early preventive measures to protect human health and reduce emissions^5^. Through the efforts of many scholars, a large number of air quality time series prediction methods have been obtained^6–8^. For example, Singh introduced Convolutional Neural Networks to correct the deviation in his research, and combined with Partial Convolutional Neural Networks to implement spatial interpolation, to improve the accuracy of $PM_{2.5}$ prediction^9^. At the same time, Qian opened up a new path for the prediction of air quality index by integrating multi model prediction and intelligent weight optimization strategy, and further promoted the improvement of prediction efficiency^10^. At present, the common prediction methods are mainly based on mathematical models, machine learning technology and intelligent optimization algorithm. Among them, spatial quantile regression analysis is a common method, which solves the problems of spatial autocorrelation and spatial heterogeneity by considering the heterogeneity of meteorological, topographic and other influencing factors^11^. In order to improve the prediction performance, geographical location, transportation, meteorological conditions and other parameters are usually used as exogenous inputs of the model^12^. Linear and nonlinear methods such as autoregressive integral moving average (ARIMA)^13^ and support vector machine (SVM)^14^ are widely used in this field. In complex cases, the combination of appropriate models can obtain better prediction results. When the influence of trend and seasonality is removed from the data, the data is decomposed into subsequences by wavelet transform, and the appropriate ARIMA model is applied, which can effectively improve the accurate prediction ability of the model for particulate pollution^15^. Although time series technology is suitable for linear data analysis, it is not suitable for atmospheric environmental data with mixed linear and nonlinear data^16^. Therefore, combining the advantages of linear models such as ARIMA with nonlinear models can generate more flexible and nuanced predictions^17^. In order to represent the complex behavior of heterogeneous time series data sets, the generalized autoregressive conditional heteroscedasticity method is used to integrate the separate prediction of ARIMA and SVM models to overcome the problem of conditional heteroscedasticity that may exist in the traditional hybrid model, and the prediction result is better than that of a single model^18^.

The mathematical model can capture the temporal correlation well, but it cannot deal with the spatial correlation, and it has strict requirements for data and high computational complexity^19^. Compared with the methods based on mathematical models, the progress of intelligent technologies and algorithms such as machine learning has opened up a very promising future for statistical methods^20^. The methods including long-term and short-term memory network (LSTM)^21^, gating recurrent unit (GRU)^22^, bidirectional long-term and short-term memory network (Bi-LSTM)^23^, convolutional neural network (CNN)^24,25^ are commonly used in deep learning. Although the single machine learning prediction model has been proved to be effective in predicting the concentration of air pollutants, more and more researchers build hybrid models to improve the prediction performance of the model under different data conditions^26^. For example, ARIMA model is used to extract and predict the linear part of air pollution, while the nonlinear part of the output is predicted by deep learning model^27^. In most cases, the change of pollutants is not only related to time, but also related to space^28^. In geostatistics, nonlinear spatial dependence is one of the important factors, and local consideration of different effects may improve the performance of the model^29^. The combination of one-dimensional convolutional neural network (1D CNN) and long-term and short-term memory (LSTM) for spatial and temporal correlation feature extraction has better performance than typical machine learning algorithms and classical deep learning models^30^. When dealing with complex time series data, the new hierarchical depth neural network HLNet shows excellent performance with its unique hierarchical design. This design enables HLNet to flexibly handle different levels of tasks or features, thus effectively improving the convergence speed of the model^31^. PhILNet is an extension, modification and improvement of HLNet architecture, focusing on improving the efficiency of single variable multi-step time series prediction (TSF) neural network, especially in the face of complex tasks such as large-scale model training, multi scene prediction and a large number of superparameter adjustments, its advantage of convergence speed is particularly significant^32^.

With the increase of the number of parameters in the network training process, the computational costs increase. The higher the dimension of the parameter space, the more complex the training process is. The complexity makes the network more likely to fall into local optimization, which leads to the longer training process or the realization of over fitting network^33^. In order to solve the problems of local optimization and parameter optimization in the deep learning model, some new models combining neural network and superparametric optimization were developed to improve the prediction performance of air quality^34^, including particle swarm optimization SVM (PSO-SVM)^35^, attention recursive neural network based on spatio-temporal map and gray wolf optimization algorithm (GWO-GART)^36^. However, the prediction of air quality related data in different regions or in the absence of data, combined with intelligent optimization algorithm, may lead to over fitting of the model and fall into local optimal solution, thus affecting the performance of the prediction model.

From the above description, it can be observed that:

- Univariate prediction is simple, intuitive and easy to explain compared with multivariate prediction. Through univariate prediction, the focus can be placed on the change trend of a specific air quality index, reduce the complexity of the model and training costs, and make it easier to understand and use the prediction results.
- Existing models tend to have high prediction ability for data in specific locations, but their robustness and generalization ability are relatively poor. Therefore, improving the generalization ability of the model can reduce the possibility of errors in practical application, reduce the repeated work time and resource cost, and improves the prediction efficiency.
- Many research articles will use a variety of algorithms to predict air quality data, and compare the advantages and disadvantages of the algorithms. However, few articles consider multiple algorithms together. Each algorithm has its own advantages, so it is important to fully utilize the prediction results of different algorithms to make comprehensive decision, which can improve the accuracy of air quality data prediction.

The fusion model proposed in this paper is based on CNN, GRU and Bi-LSTM models, aiming to solve some shortcomings of the above research. Firstly, it uses the deep learning model to predict the data of different time lengths. Secondly, Dempster Shafer (D–S) evidence theory is used to allocate the weight of the preliminary prediction. The main contributions of this paper are as follows:

1. The real data set is constructed, and five indicators of three cities in China are collected, covering a wide range of samples and observation points. By integrating an enhanced K-means algorithm, the outliers in the original data are effectively identified and corrected, and the prediction accuracy of the model is significantly improved.
2. According to the time length, the data are divided into short-term, medium-term and long-term, and MLP, RNN, CNN, LSTM, BI-LSTM and GRU models for air quality time series prediction are established respectively. By comparing the performance indicators of the six models, three models for air quality time series prediction with the best prediction results corresponding to different time scales are selected.
3. A fusion model of air quality time series prediction based on D–S evidence theory is established. Taking the prediction results and reliability of the three depth algorithm models as the three evidence bodies of the theory, and combining with D–S evidence theory to calculate the basic probability distribution, the fusion weight is further calculated. Finally, the final prediction results are obtained by fusing the prediction results of each model.

Section “Methodology” of this paper introduces the theory of deep learning algorithm and D–S evidence theory. Section 3 preprocessed the air quality time series data and established six deep learning algorithm models. Based on the performance indicators of the models, the best model suitable for predicting different time lengths was selected, and the D-S evidence theory was combined to obtain the final fusion model. In section “Results and discussion”, the fusion model is applied to air quality time series prediction, and the prediction results of the fusion model and existing algorithms are evaluated. Section “Conclusion” summarizes the core results of the study, and points out the advantages of the model and prospects for future work.

## Methodology

### Deep learning algorithms

#### Convolutional neural network

Convolutional neural network is a deep learning model specially used to deal with data with similar grid structure. CNN extracts the features in the input data through the convolution layer and pooling layer and performs the prediction task through the full connection layer. CNN can automatically learn features with translation invariance, which is suitable for local pattern recognition and helps to reduce over fitting.

**Fig. 1:**
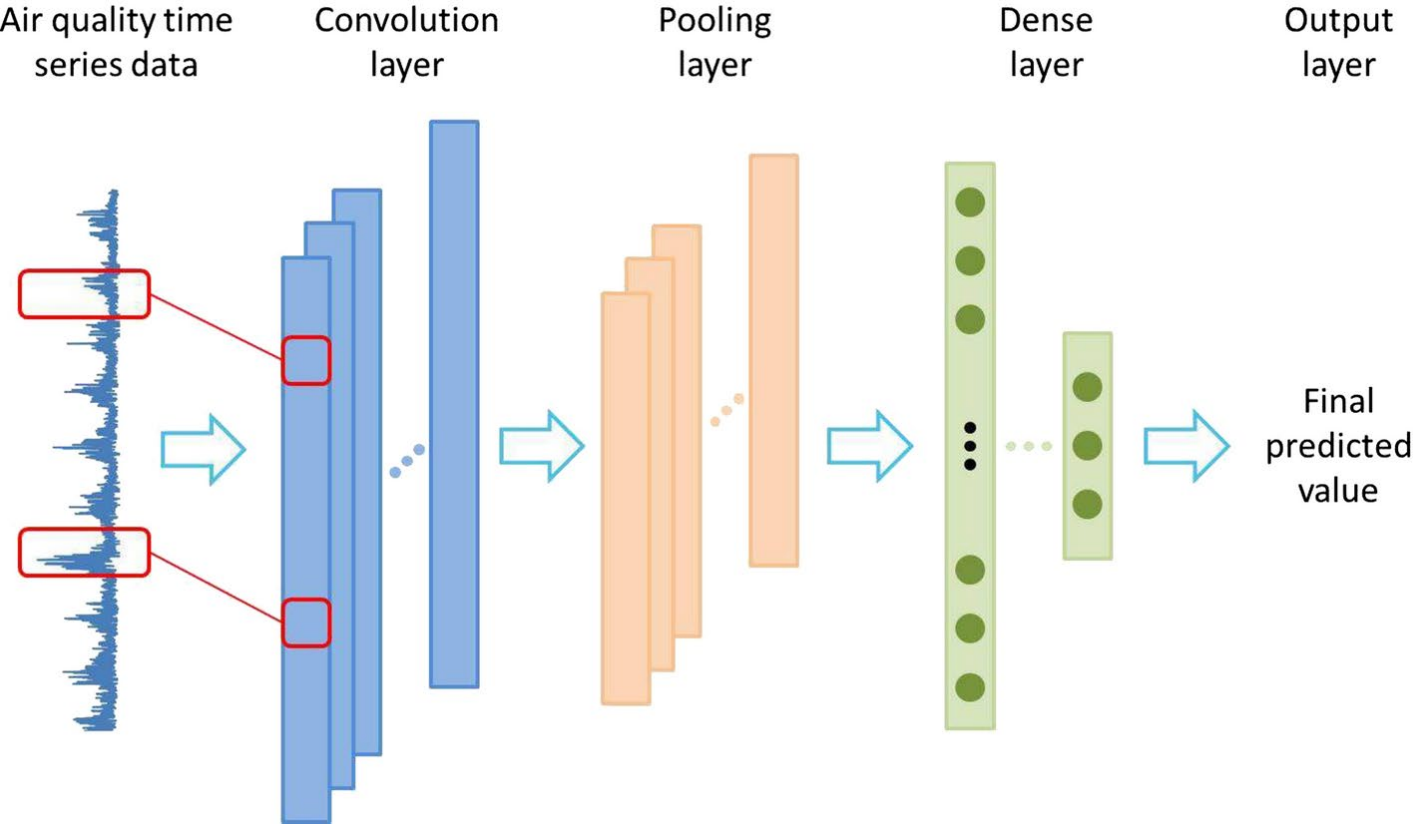
Schematic diagram of CNN structure.

In the prediction of time series data, the input of CNN model is composed of the sliding window of time series data. Local features are extracted from the convolution layer, and the pooled layer is used to down sample the output of the convolution layer, so as to reduce the data dimension and prevent over fitting^37^, and finally predict through the full connection layer.The CNN structure is shown in Fig. 1.

CNN can effectively capture local features and patterns in time series data. It has powerful nonlinear feature extraction ability and can extract important information in the data through convolution operation, such as peak value, rising or falling trend, etc.^38^. Therefore, CNN model shows good performance in short-term time series prediction task.

#### Bidirectional long short term memory

Bidirectional long short term memory is a variant of long short term memory network, which has hidden layer states in both forward and backward directions. It is widely used in the processing of sequence data, which can better capture the long-term dependencies in sequence data and provide more comprehensive context information.

In the prediction of time series data, the input of Bi-LSTM model includes the data $x_t$ of time t and the hidden state $h_{t-1}$ of time t − 1, as well as the input sequence of back propagation. For each time step, the output of Bi-LSTM consists of the hidden node output $y_t$ of the current time step and the hidden state $h_t$ passed to the next node. In the Bi-LSTM model, the forgetting gate controls the degree of forgetting past information, the input gate determines the input amount of new information, and the output gate adjusts the generated hidden state. The specific algorithm of the model is shown in the following equation:

1 $$\begin{aligned} & f_t=\sigma (W_f\cdot [h_{t-1},x_t]+b_f) \end{aligned}$$

2 $$\begin{aligned} & \quad i_t=\sigma (W_i\cdot [h_{t-1},x_t]+b_i) \end{aligned}$$

3 $$\begin{aligned} & \quad c_t=\tanh (W_C\cdot [h_{t-1},x_t]+b_C) \end{aligned}$$

4 $$\begin{aligned} & \quad y_t=f_t\cdot y_{t-1}+i_t\cdot c_t \end{aligned}$$

5 $$\begin{aligned} & \quad o_t=\sigma (W_o[h_{t-1},x_t]+b_o) \end{aligned}$$

6 $$\begin{aligned} & \quad h_t=o_t\tanh (y_t) \end{aligned}$$

where $f_t$ is the forgetting gate state of time step t, $i_t$ is the input gate state of time step t, $c_t$ is the candidate cell state of time step t, $o_t$ is the output gate state of time step t, $w_f$ is the weight coefficient, $b_f$, $b_i$, $b_c$ are the bias constants of forgetting gate, input gate and candidate cell state respectively, $\sigma$ is the sigmoid activation function, $x_t$ and $h_{t-1}$ are the current input and the previous input respectively, $W_C$ is the weight coefficient of $c_t$, $W_i$ is the weight coefficient of input, and tanh is the activation function. Because the Bi-LSTM model has long-term and short-term memory units and two-way structure design, it can capture the forward and reverse time series data at the same time, so it can better capture the trend and periodicity of the long-term time series and model the long-term changes in the time series. Therefore, Bi-LSTM has good performance in the prediction of long-term time series.

#### Gated recurrent unit

Gated recurrent unit network is a kind of gated recurrent neural network, which is similar to LSTM but simpler, including update gate and reset gate to control the information flow. GRU shows strong memory ability and adaptability when processing sequence data. Compared with LSTM model, GRU model has fewer parameters, which greatly reduces the computational complexity and improves the computational efficiency^39^.

In time series data prediction, GRU model receives the input $x_t$ of the current time step and the hidden state $h_{t-1}$ of the previous time step, which is used to synthesize the information of past nodes. Then, the model outputs $y_t$ through the hidden node of the current time step, and updates the hidden state $h_t$, so as to transfer the hidden state to the node of the next time step. GRU model controls the update and information transmission of hidden state by introducing update door and reset door. The update gate determines how much information of previous time steps is retained, while the reset gate determines how much outdated information is discarded to adapt to the current input data. The specific algorithm of GRU model is shown in the following equation^40^.

7 $$\begin{aligned} & r_t=\sigma (w_r\cdot [h_{t-1},x_t]+b_r) \end{aligned}$$

8 $$\begin{aligned} & \quad z_t=\sigma (w_z\cdot [h_{t-1},x_t]+b_z) \end{aligned}$$

9 $$\begin{aligned} & \quad \tilde{h_t} = \tanh (w_{\bar{h}} \cdot [r_t*h_{t-1}] + x_t) \end{aligned}$$

10 $$\begin{aligned} & \quad h_t = (1-z_t) \cdot h_{t-1} + z_t \cdot \tilde{h_t} \end{aligned}$$

11 $$\begin{aligned} & \quad y_t = \sigma (w_o \cdot h_t) \end{aligned}$$

### D–S evidence theory

Dempster Shafer (D–S) evidence theory is a mathematical framework for uncertain reasoning. It is widely used in inference and decision-making in the face of incomplete or uncertain information, and meets the needs of large-scale comprehensive evidence. The theory constructs a recognition framework based on the basic event set, expands the basic event set, establishes the basic probability assignment (BPA) function, and provides the Dempster Shafer fusion rule. D–S evidence theory can not only express the degree of support for events or propositions, but also fuse uncertain information, reduce the uncertainty of multi-source information, and obtain more accurate results^41^. It has become an indispensable tool in the fields of information fusion, pattern recognition and decision analysis^42^. The relevant definitions of D–S evidence theory are as follows:

(1) Frame of discernment

The identification framework $\Omega$ is an exhaustive set containing all possible assumptions E of the problem, which are mutually exclusive. The hypothesis set $\Omega$ contains N elements, which can be used to represent all the hypothesis spaces of the problem:

12 $$\begin{aligned} \Omega = \{E_1, E_2, \ldots , E_n\} \end{aligned}$$

The subset A of $\Omega$ is a proposition. The power set of $\Omega$ is $2^{\Omega }$, which is composed of all subsets of $\Omega$ and contains $2^{n}$ elements. $2^{\Omega }$ can be expressed as a set of all possible subsets of $\Omega$:

13 $$\begin{aligned} 2^{\Omega } = \{\emptyset , \{E_1\}, \{E_2\}, \ldots , \{E_n\}, \{E_1,E_2\}, \ldots , \{E_1,E_2,\ldots ,E_n\}, \ldots , \Omega \} \end{aligned}$$

(2) Basic probability assignment (BPA) function

The Basic Probability Assignment (BPA) function is a mapping from $2^{\Omega }$ to [0,1], denoted as m:$2^{\Omega }$ $\rightarrow$[0,1], which must satisfy the following two conditions:

14 $$\begin{aligned} m(\varnothing ) = 0 \quad \text {and} \quad \sum _{A \subseteq \Omega } m(A) = 1 \end{aligned}$$

where $\varnothing$ represents the empty set, and the value of m(A) represents the degree of support that the evidence has for proposition A.

(3) Belief function and plausibility function

The belief function is used to describe the degree to which evidence supports a hypothesis. The belief function of BPA is defined as follows:

15 $$\begin{aligned} \text {Bel}(A) = \sum _{B \subseteq A} m(B) \end{aligned}$$

where Bel(A) represents the overall level of belief in A. The likelihood function is used to describe the probability of observing specific evidence given a certain hypothesis. The plausibility function of BPA is defined as follows:

16 $$\begin{aligned} \text {Pl}(A) = \sum _{B \cap A \ne \varnothing } m(B) \end{aligned}$$

(4) Dempster–Shafer combination formula

For different independent evidence sources, all evidence sources have their own basic probability distribution functions. Dempster Shafer synthesis formula synthesizes these different basic probability distribution functions into a new basic probability distribution function through orthogonal sum. The formula is defined as follows:

17 $$\begin{aligned} m(A) = \frac{1}{1-k} \sum _{A_1 \cap A_2 \cap A_3 \ldots = A} m_1(A_1) m_2(A_2) m_3(A_3) \ldots \end{aligned}$$

18 $$\begin{aligned} k = \sum _{A_1 \cap A_2 \cap A_3 \ldots = \varnothing } m_1(A_1) m_2(A_2) m_3(A_3) \ldots = 1 - \sum _{A_1 \cap A_2 \cap A_3 \ldots = A} m_1(A_1) m_2(A_2) m_3(A_3) \ldots \end{aligned}$$

where k is the conflict coefficient, which is an index to measure the degree of objective difference, complexity and conflict with substantial impact caused by factors such as inconsistent information, observation error and subjective judgment. When k is close to 1, it means that the information from different evidence sources is independent and the conflict is high. In order to reduce the value of k, we can take strategies to improve the quality of evidence, such as improving the observation accuracy and optimizing the data preprocessing process.

## A fusion model of D–S evidence theory and multiple deep learning frameworks

**Fig. 2:**
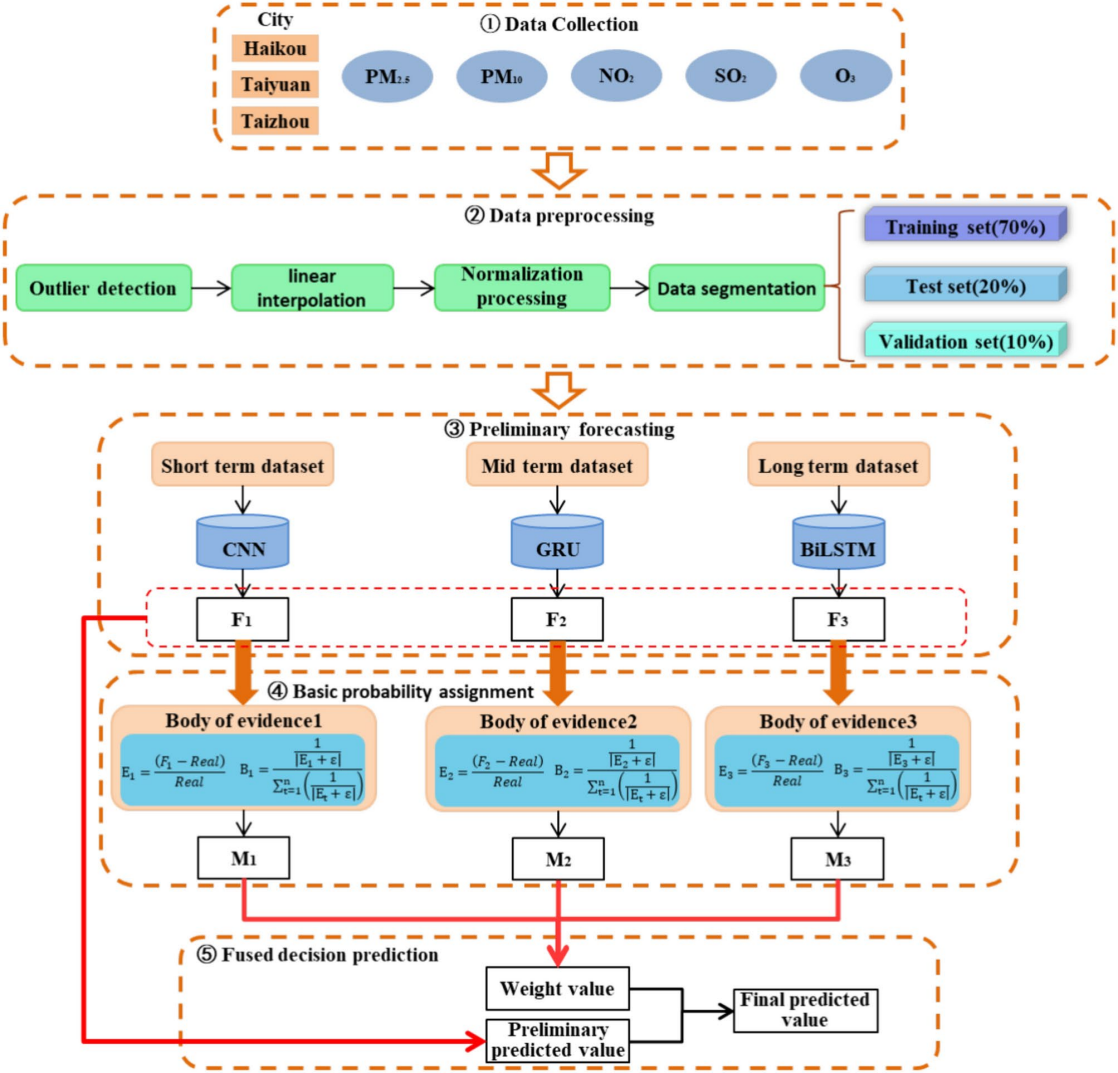
Structure diagram of air pollutant concentration prediction method.

The prediction process of the fusion model includes three steps, as shown in Fig. 2. Details are as follows:

Step 1: Data collection. Collect $PM_{2.5}$ and other data related to air quality in Haikou, Taiyuan and Taizhou, and construct the data set.

Step 2: Data preprocessing. The optimized k-means algorithm is used to detect and delete outliers in all data sets, and the linear interpolation method is used to correct these outliers. Then, the data is normalized and divided into training set, verification set and test set according to the ratio of 7:2:1, in order to improve the quality and adaptability of the data. Finally, according to the size of the input window, the processed data is further divided into short-term, medium-term and long-term data sets.

Step 3: Model selection and preliminary prediction. The six deep learning algorithms are compared, and three deep learning algorithms are selected to establish three air quality time series prediction models. The short, medium and long-term time series data are predicted respectively, and the preliminary prediction results are obtained.

Step 4: Basic probability assignment. Through the error of the initial prediction results and the reliability coefficient of the model at this time, the global probability distribution is calculated as the final weight of the fusion model.

Step 5: Integrate decision prediction. The weight is used to synthesize the preliminary prediction results of each model to get the final prediction results.

### Data collection

**Fig. 3:**
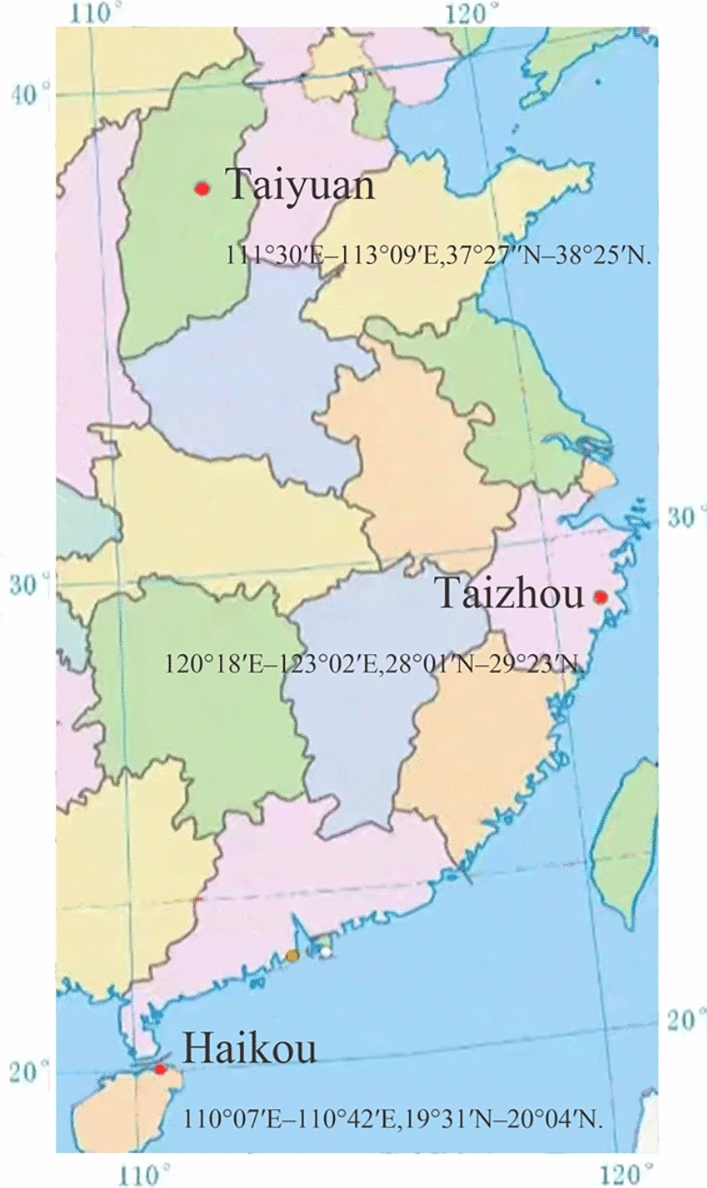
Distribution of three monitored cities. This figure is based on https://www.gov.cn/guoqing/2017-07/28/content_5043915.htm. The downloaded map GS (2019) No. 1818 was made and processed with Adobe Photoshop CC 2018, hiding some unnecessary information.

Haikou, Taizhou and Taiyuan respectively represent different types of cities such as southern cities, eastern coastal cities and northern industrial cities (Fig. 3). Haikou, located on the southern coast of Hainan Island, is a famous tourist city with good overall air quality and a tropical monsoon climate^43^. Taizhou is located in the east coast of Zhejiang Province, close to the East China Sea, bordering Wenzhou in the south, Hangzhou in the north, and facing Jinhua across the Qiantang River in the West. It has a subtropical monsoon climate. Located in the middle of North China, Taiyuan is one of the major centers of China’s energy production, chemical and metallurgical industries, and belongs to a temperate monsoon climate^44^. By studying the air quality data of various cities, the impact of different regions, climates, and economic development levels on air quality can be understood^45^. This provides a more comprehensive understanding of the issue of air pollution. Comparing the air quality data of different cities can reveal the similarities and differences between different cities. At the same time, selecting data from multiple cities for research can increase the sample size, improve the reliability and representativeness of the research, and help to find possible laws and trends. This study obtained daily air quality and related data from three cities from December 2, 2013 to August 31, 2023 through an online air quality detection and analysis platform (https://www.aqistudy.cn). The statistical properties of the data are shown in Table 1. According to the needs of different models, these data sets were converted into different input dimensions^46^, and the daily average concentrations of $PM_{2.5}$, $PM_{10}$ and other air pollutants in each city were calculated. The monitoring data from December 2013 to August 31, 2023 are used for data segmentation. The data from December 2013 to December 2022 are used as training sets for model training, while the data from January to August 2023 are used for subsequent model performance evaluation.

**Table 1:** Description of variables in the study (unit: $\upmu$g/m$^3$).

	Haikou					Taiyuan					Taizhou				
Metrics	$PM_{2.5}$	$PM_{10}$	$SO_2$	$NO_2$	$O_3$	$PM_{2.5}$	$PM_{10}$	$SO_2$	$NO_2$	$O_3$	$PM_{2.5}$	$PM_{10}$	$SO_2$	$NO_2$	$O_3$
Minimum value	2	4	2	2	12	4	6	2	5	5	2	5	0.6	2	6
Median value	14	30	11.5	11	69	45	98	20	41	84	26	46	6	20	92
Maximum value	131	167	33	65	215	377	852	429	122	296	276	358	81	89	292
Mean	18.17	33.84	5.16	12.18	75.30	56.24	109.81	37.82	43.10	93.43	31.13	53.74	7.18	21.89	93.54
Std. deviation	12.97	17.32	2.44	5.31	31.92	40.53	61.94	48.96	17.28	54.35	21.38	31.83	6.52	11.19	35.67

### Data preprocessing

#### Data outlier detection and correction

Interquartile spacing (IQR) and Z-score are common methods for detecting outliers in data sets^47^. The Z-score outlier detection method relies on the standardized distance between the data points and the mean value of the data set to quantify the degree of anomaly, especially for normal distribution data. However, its effectiveness is easily affected by extreme values, and the threshold setting is subjective. In contrast, the IQR method (interquartile distance method) sets the threshold of outliers by calculating the distance between the first quartile and the third quartile. It is robust to extreme values and is more suitable for asymmetric distribution data, but it may misjudge the normal value when dealing with heavy tailed distribution, and it is sensitive to small-scale data sets. Dynamic threshold K-means algorithm combines K-means clustering and dynamic threshold adjustment strategy, which can adaptively identify outliers according to the data distribution, not only improves the accuracy, but also provides a comprehensive perspective of data analysis with the help of its clustering ability, and has high computational efficiency, especially suitable for large-scale data sets.

K-means algorithm was first born as a vector quantization technology in the field of signal processing, and now it has become a popular tool for clustering analysis in data mining. Especially in the prediction analysis of time series data, the algorithm shows its unique value. It assigns data points to the nearest centroid and updates the centroid position through iteration to form different data clusters. Data points that do not belong to any cluster or are located at the edge of the cluster are considered outliers. In this application scenario, the algorithm divides the continuous data points in the time series into clusters with similar characteristics, so as to reveal the potential periodicity or trend, which is very important for developing the prediction model.

The core of the K-means algorithm is to minimize the within cluster sum of squares (WCSS) through the iterative process. The update formula of the cluster center (centroid) is as follows:

19 $$\begin{aligned} c_i = \frac{1}{|C_i|} \sum _{x \in C_i} x \end{aligned}$$

where $c_i$ is the center of the ith cluster, $C_i$ is the set of all data points in the ith cluster, |$C_i$| is the number of data points in cluster I, and X is a single data point. In outlier detection, it is necessary to calculate the Euclidean distance from each data point to the center of its cluster. The formula is as follows:

20 $$\begin{aligned} d(x, c_i) = \sqrt{\sum _{j=1}^n (x_j - c_{ij})^2} \end{aligned}$$

where x is the data point, $c_i$ is the center of the ith cluster, n is the feature dimension, and d(x,$c_i$) is the Euclidean distance from the data point x to the cluster center $c_i$. Outliers are usually defined as those points whose distance from the center of the data cluster to which they belong exceeds the preset threshold. The traditional static method uses a fixed threshold, which is inflexible in the face of different data distribution or noise levels, and may lead to normal points being misjudged as abnormal values, or real abnormal points being missed. In order to improve the accuracy and robustness of anomaly detection, we adopt a method to optimize the threshold, which dynamically adjusts the threshold according to the statistical characteristics of the data, so as to more accurately reflect the real situation of the data.

The selection of dynamic threshold is based on the distance distribution from data points to cluster center. The purpose of this method is to find a balance point, which can not only identify outliers that significantly deviate from most data points, but also avoid misjudging normal data points as outliers due to small fluctuations or noise in the data. By selecting the appropriate percentile (such as 95% percentile) as the threshold, we can adjust the sensitivity of anomaly detection according to the characteristics of data and analysis requirements. Specifically, first calculate the distance from all points to their corresponding cluster center, and then determine the maximum distance value corresponding to 95% of the data points in these distances as the threshold (that is, the distance between 95% of the data points and their cluster center is less than or equal to this threshold), while the remaining 5% of the data points are regarded as potential outliers. Therefore, when the distance d(x,$c_i$) from the data point to its cluster center $c_i$ is greater than the dynamically calculated threshold $threshold_dynamic$, the data point is regarded as an abnormal value. This method not only improves the accuracy of anomaly detection, but also enhances its reliability, because it can more accurately reflect the statistical characteristics of data.

**Fig. 4:**
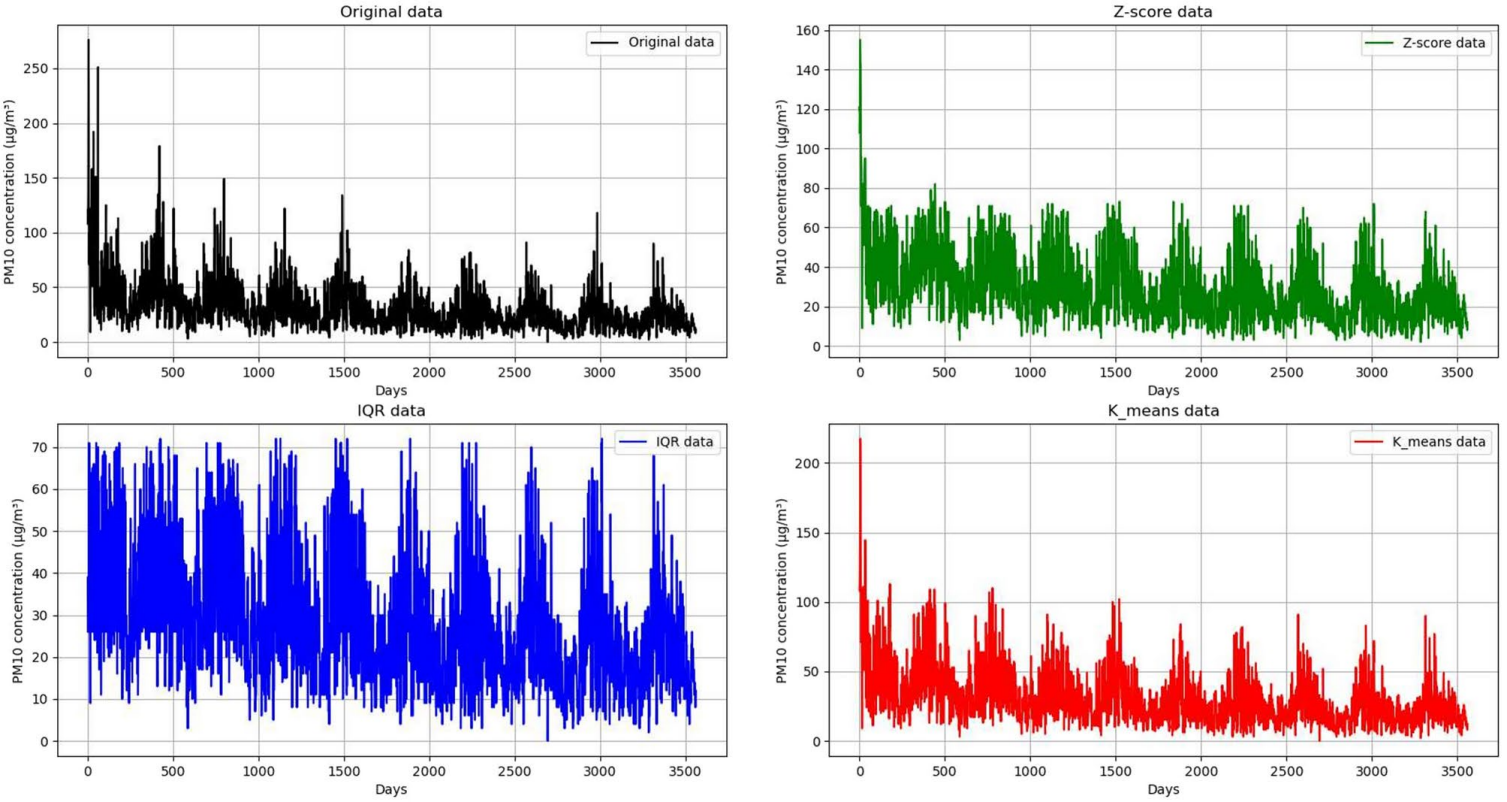
Abnormal value detection and correction of Taizhou $PM_{10}$ data.

Compared with the traditional Z-score and IQR methods, the dynamic threshold K-means algorithm combines K-means clustering and dynamic threshold adjustment strategy. It can adaptively identify outliers according to the data distribution, which not only improves the accuracy, but also provides a comprehensive perspective of data analysis with the help of its clustering ability, and has high computational efficiency, especially suitable for large-scale data sets. Figure 4 shows the data before and after the three data outlier detection methods and linear interpolation correction.

This paper uses three evaluation indexes to evaluate the prediction performance of the model, which have been widely used in time series prediction research^48,49^. These three indicators are mean absolute error (MAE), root mean square error (RMSE), mean absolute error (MAPE) and coefficient of determination ($R^2$). MAE is the average absolute value used to measure the difference between the predicted value and the real value. A small MAE means that the average error of the prediction is small. RMSE is the root mean square value used to measure the difference between the predicted value and the real value. A smaller RMSE indicates that the overall prediction error is small. MAPE is the percentage average absolute value used to measure the difference between the predicted value and the real value. A smaller MAPE means that the average percentage error of the forecast is small. The closer the value of $R^2$ is to 1, the better the fitting effect of the model is, that is, the independent variables in the model can well explain the changes of dependent variables; On the contrary, the closer the value of $R^2$ is to 0, the worse the fitting effect of the model is, and the weaker the explanatory ability of the independent variable to the dependent variable is. Their calculation formula is as follows^50^:

21 $$\begin{aligned} & MAE = \frac{1}{N} \sum _{i=1}^{N} \left|y_i - \hat{y}_i \right| \end{aligned}$$

22 $$\begin{aligned} & \quad RMSE = \sqrt{\frac{1}{N} \sum _{i=1}^{N} \left( \hat{y}_i - y_i\right) ^2} \end{aligned}$$

23 $$\begin{aligned} & \quad MAPE = \frac{1}{N} \sum _{i=1}^{N} \left|\frac{\hat{y}_i - y_i}{y_i} \right|\times 100\% \end{aligned}$$

24 $$\begin{aligned} & \quad R^2 = 1 - \frac{\sum _{i}(\hat{y}_i - y_i)^2}{\sum _{i}(y_i - \bar{y})^2} \end{aligned}$$

In the comparative experiment of analyzing outlier processing methods, two time series prediction models, LSTM and GRU, are used to evaluate the impact of different outlier processing methods on the performance of the model. Table  2 shows the comparison results of abnormal value processing methods for the performance evaluation of Taizhou PM pollutant prediction model.

**Table 2:** Comparison of outlier handling methods.

Data set	Outlier detection	LSTM				GRU			
		MAE	RMSE	MAPE	$R^2$	MAE	RMSE	MAPE	$R^2$
$PM_{2.5}$	Unprocessed data	9.499	13.884	0.419	0.538	9.092	13.590	0.374	0.558
	Z-score	8.728	11.954	0.367	0.450	8.413	11.176	0.399	0.519
	IQR	8.813	11.324	0.435	0.383	8.433	11.100	0.395	0.408
	K-means	9.183	12.850	0.398	0.558	8.815	12.498	0.370	0.582
	Optimize K-means	8.362	11.903	0.349	0.578	8.330	11.831	0.348	0.591
$PM_{10}$	Unprocessed data	14.899	21.556	0.347	0.510	14.707	21.493	0.334	0.513
	Z-score	13.635	18.545	0.312	0.455	14.216	19.500	0.316	0.397
	IQR	13.572	17.974	0.331	0.392	13.570	17.807	0.339	0.404
	K-means	14.359	20.250	0.327	0.529	14.348	20.177	0.328	0.533
	Optimize K-means	13.594	19.158	0.310	0.541	13.489	19.197	0.296	0.539

It can be clearly observed from the table data that the optimized K-means algorithm shows significant performance advantages in the field of outlier preprocessing. Compared with the unprocessed data and other widely used outlier processing methods, the optimized K-means algorithm can more effectively reduce the prediction error of the model, thus significantly improving the prediction accuracy of the model. This excellent performance is not only verified in the LSTM model, but also significant in the GRU model, which is embodied in the significant reduction of key model performance indicators and the corresponding improvement of the coefficient of determination. These changes clearly show that the fitting effect and prediction accuracy of the model have been substantially improved after using the optimized K-means algorithm to preprocess outliers.

After the outliers are detected and deleted by optimizing the k-means algorithm, the missing values need to be interpolated to ensure the integrity of time series data. Common data interpolation methods include cluster center replacement outliers, linear interpolation and multiple interpolation^51^. In order to determine the most suitable interpolation method for the experimental data set, in order to determine the most suitable interpolation method for the experimental data set, two time series prediction models, LSTM and GRU, are used as the evaluation benchmark, and the effects of different interpolation methods on improving the performance of the model are analyzed. As shown in Table 3, in the data correction process of the two data sets, the performance of the linear interpolation method in the model is significantly better than that of the other two methods.

**Table 3:** Comparison of data interpolation methods.

Data set	Interpolation method	LSTM				GRU			
		MAE	RMSE	MAPE	$R^2$	MAE	RMSE	MAPE	$R^2$
$PM_{2.5}$	Replace K-means outlier with cluster center	9.281	13.800	0.318	0.522	9.112	13.592	0.365	0.536
	linear interpolation method	8.362	11.903	0.349	0.578	8.330	11.830	0.348	0.591
	linear interpolation with cubic	8.679	11.840	0.412	0.567	8.198	11.507	0.356	0.591
$PM_{10}$	Replace K-means outlier with cluster center	14.346	21.002	0.313	0.512	14.247	20.949	0.298	0.514
	linear interpolation method	13.594	19.157	0.310	0.541	13.489	19.197	0.296	0.539
	linear interpolation with cubic	13.778	19.527	0.340	0.533	13.871	19.445	0.339	0.537

#### Data normalization and segmentation

Before inputting the data into the model, it is necessary to normalize them to eliminate the influence of data dimension on the model and accelerate the model training. The normalization formula is as follows:

25 $$\begin{aligned} X_n = \frac{{X - X_{\min }}}{{X_{\max } - X_{\min }}} \end{aligned}$$

where $X_n$ is the normalized data result, X is the original data result of the data result set, $X_{min}$ is the smallest centralized data result of air pollutant concentration statistics, and $X_{max}$ is the largest centralized data result of air pollutant concentration statistics. After normalization, the data were divided into training set (70%), validation set (20%) and test set (10%). Then, according to the length of time, it is divided into weeks (7 days), quarters (30 days) and years (365 days), forming short-term, medium-term and long-term input data. Each time length is used as the window size of the input data, that is, the data of the past 7 days, 30 days or 365 days are used as the input of the model for training each time to predict the data of the next time unit (that is, the next day).Data of different time lengths contain information of different scales. Short term data (such as 7 days) can capture the recent change trend, medium-term data (such as 30 days) can reflect the cyclical changes in a quarter, and long-term data (such as 365 days) can reveal the annual cycle and long-term trend. By combining these information, the model can understand the characteristics of time series data more comprehensively. By fusing the prediction results of the three groups of data, we can get more accurate and stable prediction results. This fusion method makes use of the information on different time scales and reduces the possible deviation of the prediction on a single time scale.

### Selection of deep learning algorithms

For short-term, medium-term and long-term time series data, it is necessary to select the appropriate prediction model. This study selected several models, including MLP, RNN, CNN, LSTM, Bi-LSTM and GRU. These models use different parameters for training and prediction, and evaluate their performance indicators by comparing their performance on the training set. Finally, the most suitable model is selected as the short-term, medium-term and long-term prediction model, and they are fused. All models use the five parameters ($PM_{2.5}$, $PM_{10}$, $NO_2$, $SO_2$, $O_3$) involved in the air quality index as inputs.

In the model training, the grid search method is used to select the super parameters. In MLP, RNN, LSTM, Bi-LSTM and GRU, the hidden layer size, number of layers, window size and return sequence form a super parameter space. The learning rate can be set to 0.01 or 0.001, the hidden layer size can be 32, 64 or 128 neurons, and the window size can be 7, 30 or 365. In CNN, the number of convolution kernels, the size of convolution kernels, the step size and the number of convolution layers constitute the hyper parameter space. The number of convolution kernels is 16, 32 or 64, the size of convolution kernels is 2 $\times$ 2, 3 $\times$ 3, 5 $\times$ 5 or 7 $\times$ 7, the step size is 0 or 2, and the number of convolution layers is 1, 2 or 3. The experimental results of all models were compared by adjusting all the super parameters. Tables 4, 5 and 6 respectively show the best hyper parameters found for each model in the $PM_{2.5}$, $PM_{10}$ and $NO_2$ data sets of each city.

**Table 4:** The best hyperparameters found by each model in the $PM_{2.5}$ dataset of three cities.

City	Enter window size	LSTM		CNN			GRU		BiLSTM	
		Layers	Units	Conv layers	Kernel sizes	Pool sizes	Layers	Units	Layers	Units
Haikou	7	2	64	32	3	2	1	128	2	128
	30	4	128	32	3	2	1	128	2	128
	365	2	64	32	5	2	4	64	2	64
Taiyuan	7	4	128	16	7	2	2	128	4	128
	30	4	128	64	7	2	4	128	4	128
	365	4	64	32	5	0	4	64	2	128
Taizhou	7	2	64	32	5	0	1	64	2	128
	30	4	128	16	3	0	2	64	2	64
	365	4	128	32	2	2	2	64	1	128

**Table 5:** The best hyperparameters found by each model in the $PM_{10}$ dataset of three cities.

City	Enter window size	LSTM		CNN			GRU		BiLSTM	
		Layers	Units	Conv layers	Kernel sizes	Pool sizes	Layers	Units	Layers	Units
Haikou	7	2	64	32	5	0	1	64	2	64
	30	4	128	16	7	2	2	64	2	64
	365	4	128	64	3	2	1	64	2	128
Taiyuan	7	4	64	64	3	0	4	128	2	128
	30	2	128	64	3	2	4	128	4	64
	365	2	128	64	7	0	1	32	4	64
Taizhou	7	4	64	64	3	0	4	128	2	128
	30	2	128	16	3	0	1	128	2	64
	365	4	128	16	7	2	1	64	2	128

**Table 6:** The best hyperparameters found by each model in the $NO_2$ dataset of three cities.

City	Enter window size	LSTM		CNN			GRU		BiLSTM	
		Layers	Units	Conv layers	Kernel sizes	Pool sizes	Layers	Units	Layers	Units
Haikou	7	4	128	32	3	0	4	64	2	128
	30	4	128	64	3	0	4	64	2	128
	365	2	128	32	7	2	2	64	4	128
Taiyuan	7	4	128	16	3	0	1	64	2	32
	30	2	128	64	3	0	2	32	2	64
	365	4	128	64	7	2	1	32	2	128
Taizhou	7	4	64	16	3	0	4	128	2	64
	30	4	128	32	5	0	1	32	2	128
	365	4	128	16	7	2	1	32	2	128

In general, from the perspective of model and data set, there is no obvious rule to be found, which indicates that more layers or units need to be added to improve the performance of the model. The optimal parameters of each model obtained by grid search method can improve the performance of the model, help us choose the appropriate model complexity, avoid over fitting and under fitting, so as to improve the generalization ability and interpretability of the model, and provide the basis for model selection.

First of all, the research on short-term prediction shows that CNN has shown excellent performance in processing the data set of five parameters involving the air quality index of three provinces. The number of instances of six deep learning algorithms (MLP, RNN, CNN, LSTM, GRU, Bi-LSTM) whose architecture is superior to all other architectures in short-term prediction is 1, 1, 21, 10, 7 and 5, respectively. CNN has significantly better prediction ability. In addition, for the three performance indicators (MAE, MSE and MAPE), CNN has improved the performance by 0.60%, 0.85% and 1.44% on average compared with the sub optimal architecture. Therefore, CNN is the best choice for short-term prediction. Table 7 shows the best model performance indicators obtained during the training of each model when the input window is 7. In Table 7 - 10, the values marked in bold represent the best performance of the six models in each performance index.

**Table 7:** MAE, MSE, and MAPE for each model when the input window size is 7.

Model	Performance index	Haikou					Taiyuan					Taizhou				
		$PM_{2.5}$	$PM_{10}$	$SO_2$	$NO_2$	$O_3$	$PM_{2.5}$	$PM_{10}$	$SO_2$	$NO_2$	$O_3$	$PM_{2.5}$	$PM_{10}$	$SO_2$	$NO_2$	$O_3$
MLP	MAE	3.839	7.737	2.210	0.571	14.84	15.78	25.92	9.127	4.152	21.62	6.883	11.41	4.203	0.864	18.70
	RMSE	5.695	8.157	3.190	0.967	20.16	22.37	44.09	11.70	6.228	28.96	10.01	16.60	5.907	2.375	24.12
	MAPE	0.283	0.223	0.262	0.123	0.196	0.506	0.381	0.284	0.366	0.258	0.401	0.323	0.236	**0.118**	0.259
RNN	MAE	3.878	6.141	2.198	0.580	14.85	15.71	25.77	9.143	4.299	21.78	6.975	11.59	4.226	0.905	18.71
	RMSE	5.676	8.364	3.194	0.972	20.10	22.50	44.00	11.71	6.236	29.00	10.03	16.69	5.875	2.426	24.30
	MAPE	0.300	0.260	0.252	0.128	0.196	0.489	0.385	0.291	0.420	0.275	0.419	0.337	0.243	0.134	**0.251**
CNN	MAE	**3.795**	**5.666**	**2.122**	**0.537**	**14.59**	**15.41**	**25.49**	**8.597**	4.144	**21.43**	6.806	**11.03**	**4.150**	0.875	18.62
	RMSE	**5.661**	**8.060**	**3.144**	0.954	**19.90**	22.07	43.46	**11.43**	6.247	28.82	10.07	**16.40**	**5.833**	2.546	24.08
	MAPE	**0.275**	0.228	0.241	**0.109**	0.192	0.480	0.383	0.277	0.368	**0.255**	0.371	0.293	0.238	0.125	0.258
LSTM	MAE	3.827	5.699	2.151	0.541	14.67	15.48	25.53	9.135	**4.118**	21.47	6.792	11.19	4.188	0.847	18.61
	RMSE	5.707	8.180	3.181	0.955	20.27	**22.07**	**43.19**	11.66	**6.197**	**28.73**	**9.980**	16.61	5.872	**2.337**	24.01
	MAPE	0.280	**0.219**	0.243	0.114	0.188	0.480	**0.372**	**0.277**	0.359	0.256	0.381	0.300	0.235	0.126	0.261
GRU	MAE	3.820	5.697	2.163	0.538	14.60	15.48	25.60	9.058	4.143	21.50	**6.732**	11.18	4.173	**0.843**	**18.56**
	RMSE	5.708	8.125	3.217	**0.952**	20.06	22.29	43.35	11.62	6.204	29.01	9.993	16.59	5.857	2.355	**23.99**
	MAPE	0.278	0.225	0.238	0.112	0.189	0.484	0.383	0.279	0.363	0.259	0.370	0.294	0.237	0.122	0.258
BiLSTM	MAE	3.850	5.704	2.163	0.547	14.75	15.51	26.15	9.070	4.288	21.79	6.756	11.28	4.174	0.890	18.66
	RMSE	5.743	8.177	3.201	0.970	20.42	22.21	44.23	11.59	6.361	29.11	10.11	16.79	5.861	2.402	24.09
	MAPE	0.281	0.224	**0.237**	0.112	**0.187**	**0.476**	0.382	0.287	**0.355**	0.259	**0.365**	**0.292**	**0.233**	0.122	0.257

Secondly, for the medium-term prediction, the number of instances in which the architecture of the six deep learning algorithms is superior to all other architectures is 0, 2, 9, 7, 10 and 17, respectively. Meanwhile, the performance of GRU is increased by 0.88%, 0.26% and 1.32% compared with the sub optimal architecture, which further verifies the good performance of GRU in the medium-term prediction. Table 8 shows the best model performance indicators obtained during the training of each model when the input window is 30.

**Table 8:** MAE, MSE, and MAPE for each model when the input window size is 30. Significant values are in bold.

Model	Performance index	Haikou					Taiyuan					Taizhou				
		$PM_{2.5}$	$PM_{10}$	$SO_2$	$NO_2$	$O_3$	$PM_{2.5}$	$PM_{10}$	$SO_2$	$NO_2$	$O_3$	$PM_{2.5}$	$PM_{10}$	$SO_2$	$NO_2$	$O_3$
MLP	MAE	3.810	5.738	2.199	0.566	15.03	16.02	27.27	9.023	4.180	21.54	6.802	11.28	4.245	0.986	19.04
	RMSE	**5.632**	8.153	3.199	0.970	20.29	22.50	43.88	11.58	6.261	29.03	9.909	16.49	5.840	2.612	24.41
	MAPE	0.279	0.228	0.251	0.120	0.199	0.521	0.441	0.287	0.375	0.272	0.390	0.315	0.255	0.148	0.272
RNN	MAE	3.928	6.076	2.262	0.554	14.83	15.83	25.95	9.205	4.301	21.52	6.734	11.64	4.266	0.968	18.97
	RMSE	5.667	8.257	3.214	0.973	**20.21**	22.38	43.86	11.80	6.391	28.57	9.916	16.64	5.843	2.631	24.12
	MAPE	0.308	0.257	0.272	0.114	0.194	0.496	0.397	0.284	0.405	0.263	0.376	0.347	0.256	0.144	0.268
CNN	MAE	3.890	5.742	**2.148**	0.546	14.76	**15.31**	26.07	9.035	4.192	21.38	6.703	11.27	**4.132**	0.960	18.64
	RMSE	5.661	8.153	3.194	0.954	20.26	22.10	44.05	11.56	6.170	**28.44**	**9.812**	16.58	5.816	2.835	23.98
	MAPE	0.282	0.223	**0.236**	0.113	**0.182**	**0.458**	0.390	**0.279**	0.397	0.261	0.387	0.308	0.231	0.129	0.262
LSTM	MAE	**3.804**	**5.673**	2.166	**0.541**	14.66	15.40	26.03	9.003	4.183	21.51	**6.675**	11.11	4.210	0.915	18.59
	RMSE	5.688	8.088	3.210	**0.947**	20.38	**21.94**	43.39	11.56	6.112	28.74	9.845	16.41	5.867	2.551	23.92
	MAPE	0.281	0.221	0.239	0.115	0.185	0.464	0.411	0.288	0.368	0.273	0.370	**0.304**	0.246	0.125	0.266
GRU	MAE	3.818	5.712	2.163	0.543	**14.37**	15.34	**25.26**	**8.962**	**4.100**	21.32	6.787	11.26	4.207	0.940	**18.52**
	RMSE	5.673	8.109	**3.189**	0.956	20.23	22.07	43.64	**11.52**	**6.093**	28.77	9.892	**16.36**	**5.806**	2.731	**23.82**
	MAPE	**0.278**	**0.215**	0.240	**0.112**	0.199	0.475	**0.382**	0.286	0.368	**0.257**	0.378	0.312	**0.228**	0.123	0.259
BiLSTM	MAE	3.811	5.744	2.171	0.550	14.99	15.36	25.55	8.967	4.137	**21.30**	6.700	**11.09**	4.225	**0.870**	18.59
	RMSE	5.686	**8.054**	3.208	0.953	20.58	22.03	**43.22**	11.53	6.155	28.47	9.887	16.40	5.823	**2.398**	23.96
	MAPE	0.282	0.228	0.239	0.115	0.190	0.473	0.386	0.285	**0.357**	0.269	**0.368**	0.305	0.253	**0.122**	**0.258**

Finally, for long-term prediction, the study found that Bi-LSTM is an effective model selection. The number of instances in which the six deep learning algorithms outperform all other architectures in the long-term prediction is 1, 2, 8, 5, 21 and 8, respectively. On each dataset, Bi-LSTM has improved the performance by 0.60%, 0.57% and 0.68% compared with the sub optimal architecture. Table 9 shows the best model performance indicators obtained during the training of each model when the input window is 365.

**Table 9:** MAE, MSE, and MAPE for each model when the input window size is 365. Significant values are in bold.

Model	Performance index	Haikou					Taiyuan					Taizhou				
		$PM_{2.5}$	$PM_{10}$	$SO_2$	$NO_2$	$O_3$	$PM_{2.5}$	$PM_{10}$	$SO_2$	$NO_2$	$O_3$	$PM_{2.5}$	$PM_{10}$	$SO_2$	$NO_2$	$O_3$
MLP	MAE	4.171	6.442	2.489	0.632	16.72	17.63	29.98	10.22	4.382	23.46	7.421	12.87	5.093	1.504	21.42
	RMSE	5.941	8.834	3.541	1.040	21.55	25.18	47.02	13.01	6.230	30.49	10.57	17.87	6.852	3.525	26.90
	MAPE	0.331	0.272	0.269	0.131	0.235	0.483	0.475	0.331	0.422	0.278	0.430	0.390	0.299	0.228	0.303
RNN	MAE	3.898	5.838	2.323	0.587	14.77	15.99	26.96	9.250	4.619	21.88	6.784	11.27	4.296	0.897	18.55
	RMSE	5.679	8.251	3.256	0.985	20.07	22.61	44.16	11.79	6.543	28.91	9.871	16.59	6.022	2.477	23.99
	MAPE	0.303	0.224	0.290	0.128	0.197	0.510	0.428	**0.278**	0.458	0.267	0.388	0.307	**0.229**	0.128	0.261
CNN	MAE	3.819	**5.623**	2.209	**0.544**	14.80	15.39	25.96	8.957	4.142	21.08	6.701	11.04	4.166	1.245	18.64
	RMSE	5.693	**8.056**	3.192	0.964	20.63	22.03	43.72	**11.48**	6.186	28.29	9.760	16.36	5.835	3.074	24.13
	MAPE	0.279	0.223	0.262	**0.112**	**0.182**	0.467	0.398	0.281	**0.351**	0.263	0.395	0.312	0.235	0.190	**0.249**
LSTM	MAE	3.814	5.702	2.166	0.547	14.86	15.53	26.33	9.002	4.157	21.48	6.666	**11.01**	4.263	0.861	18.57
	RMSE	5.708	8.084	3.198	0.965	20.35	22.17	44.69	11.59	6.131	28.66	9.820	**16.24**	5.819	2.437	23.92
	MAPE	**0.272**	0.228	**0.245**	0.114	0.189	0.488	0.398	0.288	0.370	0.261	**0.367**	**0.304**	0.261	0.130	0.261
GRU	MAE	3.818	5.746	2.177	0.546	**14.74**	15.39	25.73	**8.955**	4.107	21.27	6.695	11.18	**4.147**	**0.845**	18.57
	RMSE	5.674	8.105	3.200	0.958	20.11	22.13	43.83	11.54	6.115	28.70	9.928	16.38	5.821	**2.313**	23.96
	MAPE	0.278	0.226	0.247	0.115	0.193	**0.456**	**0.383**	0.282	0.376	**0.258**	0.369	0.316	0.235	**0.125**	0.262
BiLSTM	MAE	**3.803**	5.677	**2.145**	0.548	14.76	**15.36**	**25.39**	9.012	**4.080**	**21.01**	**6.617**	11.09	4.152	0.977	**18.49**
	RMSE	**5.667**	8.152	**3.140**	**0.957**	**20.01**	**21.95**	**43.05**	11.58	**6.037**	**28.12**	**9.744**	16.28	**5.766**	2.753	**23.78**
	MAPE	0.274	**0.221**	0.250	0.116	0.196	0.472	0.385	0.295	0.366	0.262	0.377	0.314	0.238	0.130	0.257

To sum up, through the model evaluation of the air pollutant concentration data of Haikou, Taiyuan and Taizhou, the comparison results are shown in Table 10. The brackets show the average increase between the best results and the second best results for different window sizes. It is found that CNN, GRU and Bi-LSTM models are the most competitive architectures in short-term, medium-term and long-term prediction. In time series prediction, it is observed that the number of instances of different algorithm architectures is better than that of other architectures, and the prediction ability of models in different regions will also vary greatly. Therefore, a fusion model is needed to improve the robustness and generalization ability of prediction, so as to improve the prediction efficiency.

**Table 10:** Instances where each architecture outperforms all other architectures. Significant values are in bold.

Window size	MLP	RNN	CNN	LSTM	GRU	BiLSTM
7	1 (2.54%)	1 (2.37%)	**21 (0.81%)**	10 (0.66%)	5 (0.31%)	7 (0.76%)
30	1 (0.52%)	1 (0.10%)	9 (0.90%)	7 (0.33%)	**17 (0.82%)**	10 (1.59%)
365	0 (0%)	2 (1.66%)	8 (1.72%)	6 (0.54%)	9 (1.39%)	**20 (0.63%)**

### Fusion model based on D–S evidence theory

#### Weight calculation method

Reliability plays a key role in evaluating the information quality and accuracy of prediction results. Each model has different reliability under different parameters. By evaluating the reliability of evidence sources, the importance and reliability of the model prediction information can be better assessed. According to the error $E_s$ predicted by the model, determine the reliability $B_s$ value of each model at any time point, that is, the reliability of the model at that time is reflected as follows:

26 $$\begin{aligned} & E_s = \frac{{(\hat{y}_s - y_s)}}{{y_s}} \end{aligned}$$

27 $$\begin{aligned} & \quad B_s = \frac{\frac{{1}}{{|E_s+\varepsilon |}}}{\sum _{t=1}^n \frac{{1}}{{|E_t+\varepsilon |}}} \end{aligned}$$

In order to avoid the case that the denominator prediction error $E_s$ is zero, a minimum positive number $\varepsilon$ close to zero is used. The selection of $\varepsilon$ is usually based on the consideration of numerical stability and calculation accuracy. In practical applications, the specific value of $\varepsilon$ can be determined by a variety of methods. For example, it can be set according to the noise level of the data, the accuracy requirements of the model and the limitation of computing resources. Common practices include selecting a number far less than the minimum non-zero value in the dataset, or setting a fixed small value based on the rule of thumb. In this study, according to the characteristics of the data and the needs of the prediction task, we finally determined an appropriate $\varepsilon$ value through experimental verification, in order to ensure the stability of the calculation and the accuracy of the prediction. Where $E_t$ represents the prediction error of each model and is used to accumulate the prediction error of n neural network models. $E_s$ represents the prediction error of one of the models, $y_s$ represents the actual concentration of environmental pollutants, and $\hat{y}_s$ represents the predicted concentration of environmental pollutants. Then, the prediction results and reliability coefficients of N neural network models are used as evidence to evaluate the degree of conflict between the evidences. If the conflict degree meets the requirements, the D–S evidence theory is used to fuse the prediction results of n neural network models to get the final prediction result. If the conflict degree does not meet the requirements, it is necessary to re process the historical data, re divide the training set, verification set and test set, and then continue the training until the conflict degree meets the requirements. The specific steps are as follows:

Firstly, each b prediction value of n neural network models is divided into a group, and the probability distribution corresponding to each prediction value is calculated through the probability distribution function. Let the q-th output value of the s-th neural network be $O_s$(q), then:

28 $$\begin{aligned} \left\{ \begin{aligned} m_s(q)&= \frac{O_s(q)}{\sum _{j=1}^b O_s(q)} \times B_s \\ m_s(\theta )&= 1 - B_s \end{aligned} \right. \end{aligned}$$

where $m_s$(q) is the probability distribution of the s-th neural model to the q-th output value, and $m_s$($\theta$) represents the basic probability distribution function of uncertainty $\theta$. Secondly, according to the combination formula of evidence theory, the basic probability distribution of each combined state is obtained as follows:

29 $$\begin{aligned} \left\{ \begin{aligned} m(A)&= \frac{{\sum _{\bigcap A_u=A} \prod _{s\le u\le n} m_s(A_u) }}{{1-K}}, \quad A \ne \emptyset \\ m(\emptyset )&= 0, \quad A = \emptyset \end{aligned} \right. \end{aligned}$$

where A represents the set of all output values of all models, and $A_u$ is the u-th output value or the U-Th element. If K=$\sum _{\bigcap A_u=A} \prod _{s\le u\le n} m_s(A_u) < 1$, the model is the final prediction model. If K$\rightarrow$1 or K=1, it means that the evidence is highly conflicting, and the pretreatment of the data set needs to be optimized to reduce the conflicts caused by inconsistent information. For example, re clean the data set, remove duplicate, inconsistent or wrong data information, reduce evidence conflict, and further extract features, reduce noise and redundant information in the data set, and improve the accuracy of evidence until K < 1.

#### Integrate decision prediction

The weight calculation method is obtained through D–S evidence theory, and a fusion model based on a variety of deep learning algorithms is proposed to predict the time series of air quality. The fusion model includes the preliminary prediction models of short-term, medium-term and long-term data sets, which are CNN, GRU and Bi-LSTM models respectively. By selecting different models, we can make full use of their characteristics and expression ability to capture the short-term and long-term dependencies in time series data and improve the overall performance. In the prediction process, the data of each time length (7 days, 30 days, 365 days) are used as the window size of the input data respectively, and the preliminary prediction is made through three independent models. In the fusion process, this study evaluated the reliability of each model at each time point, and evaluated it through the model prediction error. These preliminary prediction results are fused on a daily basis, that is, the predicted values at each time point are weighted by the weight values of the three models. Then, the reliability coefficients and prediction results of the three models are taken as evidence, and the degree of conflict between evidences is evaluated by using the judgment method of evidence conflict degree in D–S evidence theory. If the set requirements are met, the prediction results of the three models are fused to obtain the final prediction results. The result of fusion can be short-term time series prediction (such as predicting the data in the next week) or long-term time series prediction (such as predicting the data in the next year), depending on the time range and strategy adopted during fusion. The time length predicted in this paper is the time length of the test set.

The advantage of this fusion model is that it can make full use of the different advantages of a variety of deep learning algorithms to improve the modeling ability of time series data. By using D–S evidence theory for reliability evaluation and evidence fusion, the uncertainty of a single model can be reduced, and the reliability and stability of the prediction results can be improved^52,53^. It should be noted that the specific weight calculation and evidence fusion methods may be adjusted according to the specific problems and the characteristics of the data set, which depends on the researchers’ comprehensive consideration of the model performance and reliability. At the same time, the selection and evaluation of the model also need to be considered in combination with the actual situation.

## Results and discussion

The time series prediction fusion model was developed in this study using PyCharm software on a computer workstation. The workstation is equipped with a 7th generation Intel(R) Core(TM) i7-7700HQ CPU @ 2.80GHz and 8GB RAM, running on the Windows 10 operating system. The study utilized three individual models (LSTM^54^, HLNet, PHILNet) and five ensemble models (CNN-LSTM^30^, DS-CNN, DS-GRU, DS-BILSTM, DS-CNN-GRU-BILSTM) to predict five air quality indicators ($PM_{2.5}$, $PM_{10}$, $NO_2$, $SO_2$, $O_3$) in three different climate cities (Taizhou, Taiyuan, Haikou). Three accuracy evaluation metrics (MAE, RMSE, MAPE) were used to compare their prediction performance.

**Fig. 5:**
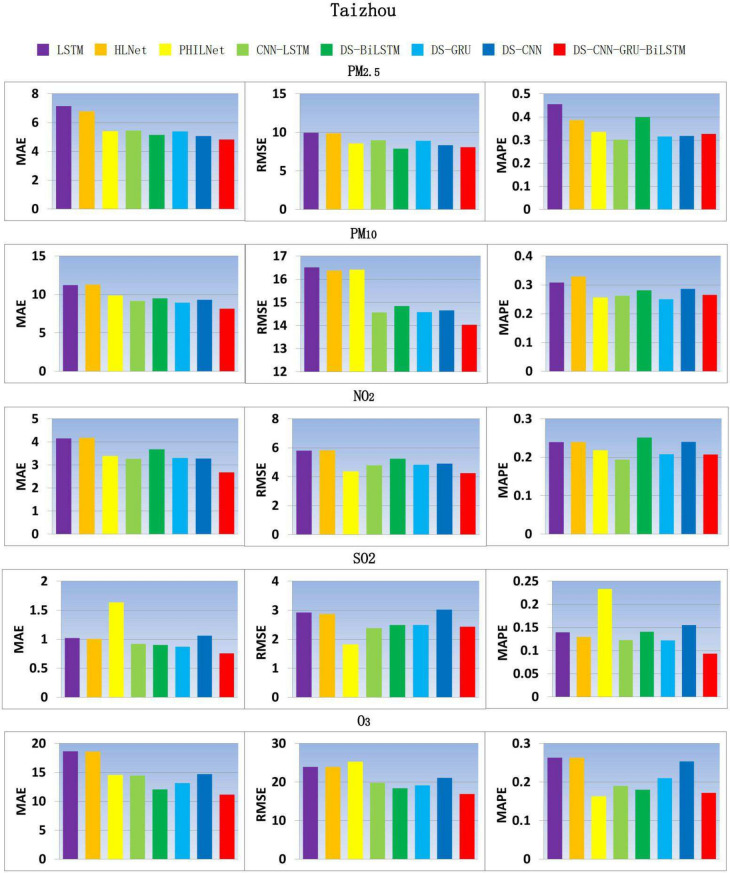
Comparison model results of air pollutant indicators in Taizhou city.

According to Fig. 5, in terms of MAE, the performance of DS-CNN-GRU-BILSTM in $PM_{2.5}$, $PM_{10}$, $NO_2$, $SO_2$ and $O_3$ was improved by 4.87%, 8.85%, 18.51%, 12.85% and 7.38% respectively compared with the sub optimal architecture; In terms of RMSE, the performance of $PM_{10}$, $NO_2$ and $O_3$ were improved by 3.74%, 2.79% and 8.13% respectively compared with the sub optimal architecture. The comparison results of all models show that the fusion model proposed in this paper performs better in prediction ability and model stability, and reduces the volatility of prediction results.

**Fig. 6:**
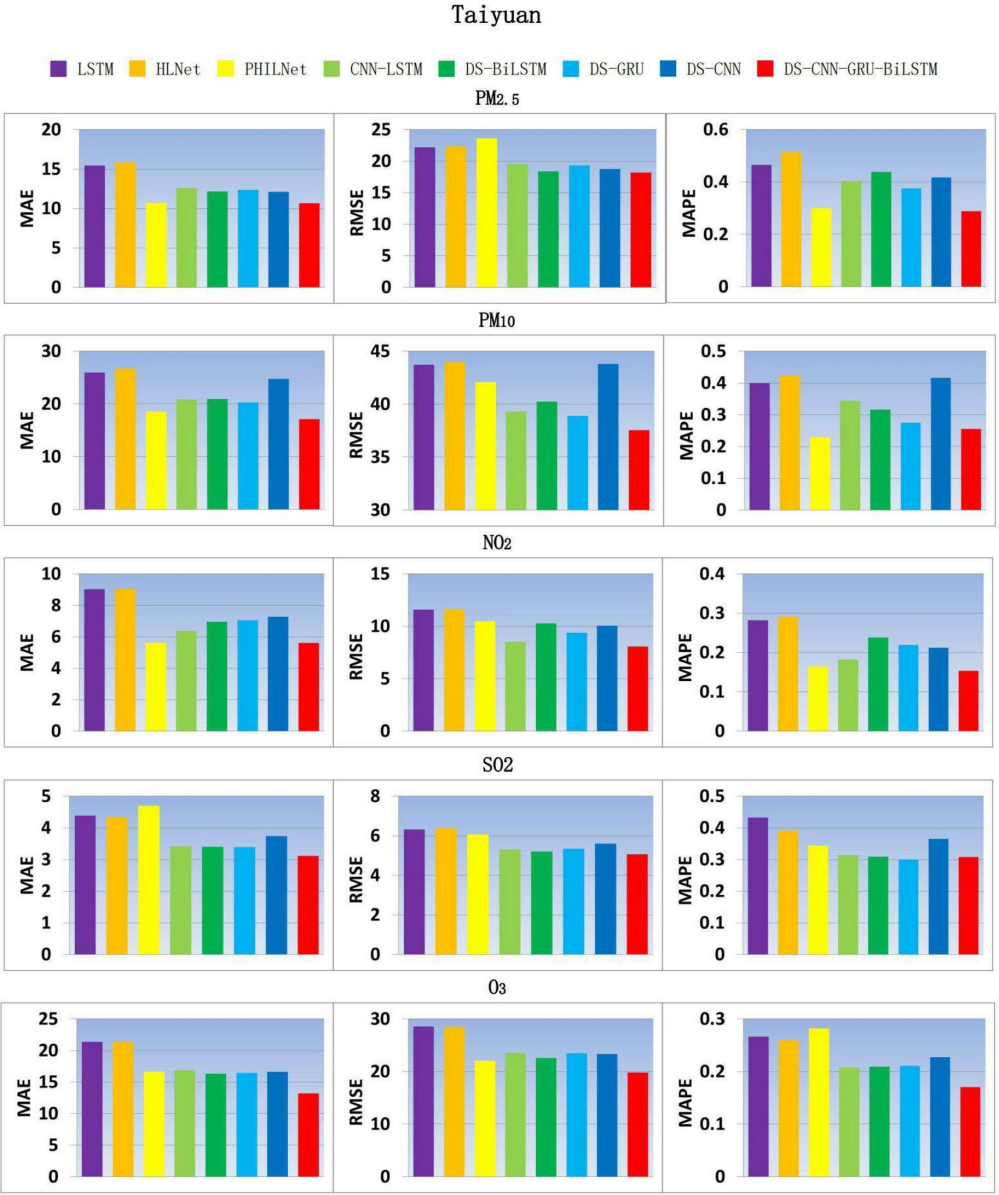
Comparison model results of air pollutant indicators in Taiyuan city.

According to Fig. 6, in terms of MAE, the performance of DS-CNN-GRU-BILSTM in $PM_{2.5}$, $PM_{10}$, $NO_2$, $SO_2$ and $O_3$ was improved by 0.77%, 7.88%, 0.11%, 8.01% and 19.10% respectively compared with the sub optimal architecture; In terms of RMSE, the performance of $PM_{2.5}$, $PM_{10}$, $NO_2$, $SO_2$ and $O_3$ were improved by 1.12%, 3.42%, 13.71%, 2.86% and 10.25% respectively compared with the sub optimal architecture; MAPE has also been improved to a certain extent, which shows that the model performs better on the data outside the training set and test set, has better generalization performance, and can better adapt to new unseen data.

**Fig. 7:**
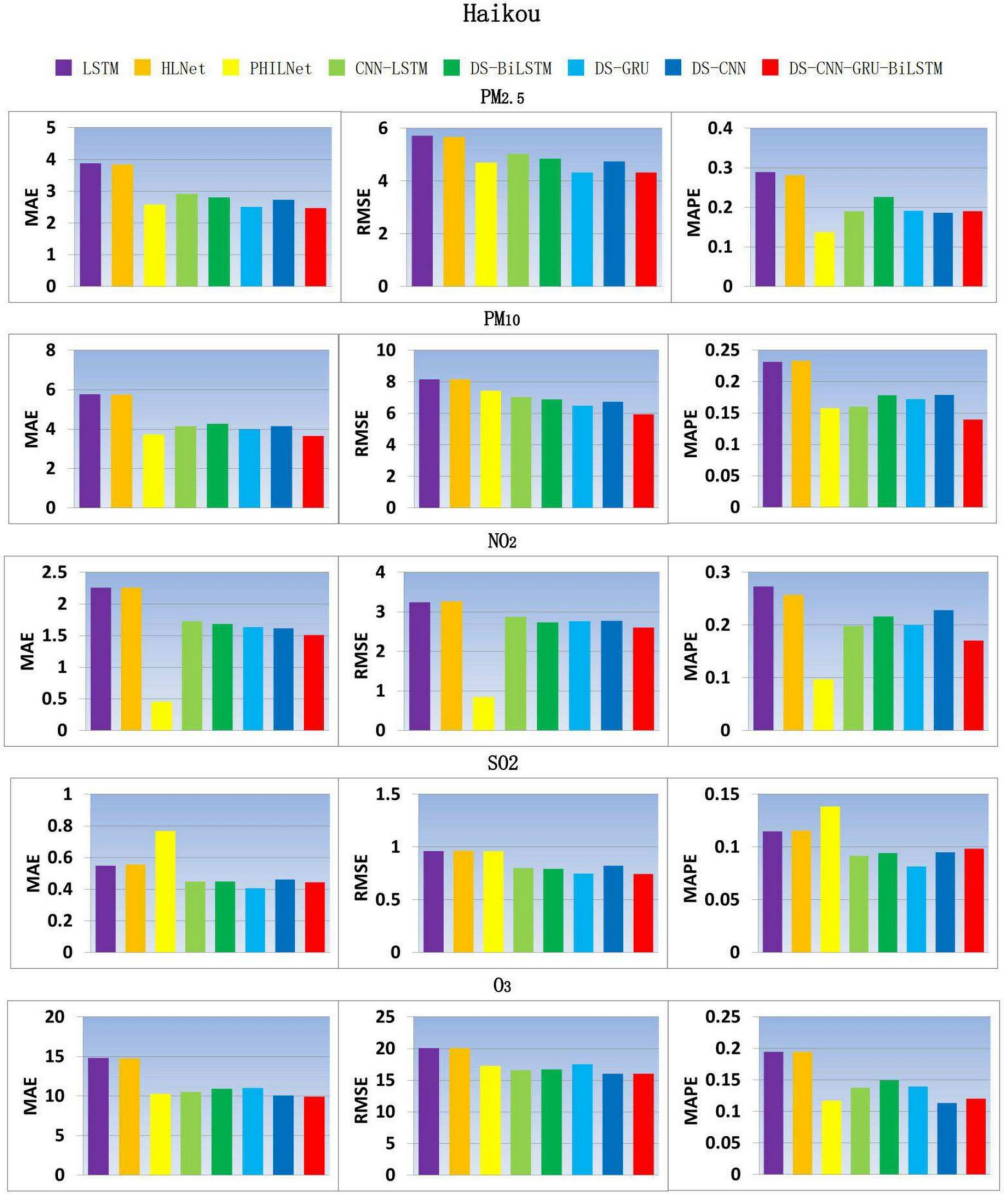
Comparison model results of air pollutant indicators in Haikou city.

According to the analysis results in Fig. 7, the DS-CNN-GRU-BILSTM model has improved in most of the model performance indicators. However, there are also some other combined models that have better prediction results than DS-CNN-GRU-BILSTM, especially in the index of average absolute percentage error. This situation may be related to the interval size of the value to be predicted in the data sample. When the variation range of air quality index in the sample is small, the variation range of relative error may also be small, which will affect the prediction performance of the model.

In general, the fusion model proposed in this paper takes into account the differences between the basic models, which make it have certain advantages over other integration methods. The experimental results show that the MAE, RMSE and MAPE of the fusion model on all data sets are reduced by 1.91%, 1.53% and 1.43%, respectively. The fusion model in this study shows good performance and stability in time series prediction, which provides a useful reference for practical application.

## Conclusion

This paper proposes an air quality time series prediction fusion model based on D–S evidence theory, which aims to solve the problems of insufficient generalization ability of existing models and single algorithm selection. By integrating the prediction results of various deep learning models, the accuracy of air quality prediction is improved. The model integrates the prediction results and reliability of three different algorithms, and obtains the final prediction results by calculating the basic probability distribution and fusion weight.The fusion model showed excellent accuracy improvement in the prediction of air pollutant concentrations in Haikou, Taiyuan and Taizhou. Compared with the suboptimal architecture, the fusion model achieves an average improvement of 7.42%, 4.25% and 12.82% in MAE, RMSE and MAPE, respectively. This result strongly proves the significant advantages of the fusion model in reducing the uncertainty of a single prediction model and enhancing the reliability and accuracy of air quality time series prediction. Compared with the traditional model, the fusion model has improved the prediction accuracy, robustness and generalization ability.

However, while this study has achieved remarkable results, it also faces some challenges and shortcomings. First of all, although we have fully considered the impact of climate factors, the important impact of other key spatial factors such as geomorphic conditions on air quality prediction has not been fully reflected in this study. In order to further reveal the inherent laws of air pollutant data, future research needs to incorporate more diversified spatial factors. Secondly, the classical D–S evidence theory has some limitations in dealing with highly conflicting evidence, which may be difficult to effectively integrate these evidence for accurate prediction. In addition, the nonlinear outlier correction method may not perform well in some complex scenarios, which needs further optimization and improvement. In order to overcome these challenges, future research can actively explore a new model combining neural network and hyper parameter optimization technology, in order to replace the current deep learning model, so as to further improve the prediction accuracy. At the same time, we can also consider introducing other advanced fusion algorithms, such as Bayesian network or fuzzy logic, to deal with the problem of highly conflicting evidence more effectively. For the correction of nonlinear outliers, we can try to use more complex machine learning algorithms or statistical methods in order to improve the correction effect. Through these innovative exploration and practice, we can continuously improve the time series prediction model of air quality, and provide more accurate and reliable prediction support for environmental protection and public health.

## Supplementary Information

Supplementary Information 1.

Supplementary Information 2.

Supplementary Information 3.

Supplementary Information 4.

## Data Availability

All data generated or analysed during this study are included in this published article and its supplementary material files.
