# Supplementary material for: Integrating D–S evidence theory and multiple deep learning frameworks for time series prediction of air quality
Source: Sci Rep. 2025 Feb 18;15:5971. doi: 10.1038/s41598-025-87935-3 (PMC11836142; doi:10.1038/s41598-025-87935-3)
Supplement: Supplementary file 2 — Supplementary Information 2. [file 41598_2025_87935_MOESM2_ESM.docx]

Data source: air quality online detection and analysis platform（ https://www.aqistudy.cn ）. By crawling and sorting out the data from the platform, we finally obtained the tables containing the air quality data of Haikou, Taiyuan and Taizhou, which are haikou_data.csv, taiyuan_data.csv and taizhou_data.csv respectively.
